# Changes in cervical elastography, cervical length and endocervical canal width after cerclage for cervical insufficiency: an observational ultrasound study

**DOI:** 10.1186/s12884-023-06071-w

**Published:** 2023-10-24

**Authors:** Meng-Hsuen Hsieh, Chie-Pein Chen, Fang-Ju Sun, Yi-Yung Chen, Liang-Kai Wang, Chen-Yu Chen

**Affiliations:** 1 Department of Obstetrics and Gynecology, MacKay Memorial Hospital, No. 92, Section 2, Zhong-Shan North Road, Taipei, 10449 Taiwan; 2 Department of Medical Research, MacKay Memorial Hospital, Taipei, Taiwan; 3 Department of Medicine, MacKay Medical College, New Taipei City, Taiwan

**Keywords:** Cervical insufficiency, Cervical elastography, Cervical length, Endocervical canal width, Cerclage

## Abstract

**Background:**

We previously demonstrated that pregnant women with a history of cervical insufficiency had a softer anterior cervical lip, shorter cervical length and wider endocervical canal in the first trimester. The aim of this study was to investigate changes in cervical elastography, cervical length, and endocervical canal width in the second trimester after cerclage, and further discuss whether these ultrasound parameters are predictive of preterm delivery.

**Methods:**

This was a secondary analysis of cervical changes in singleton pregnancies after cerclage from January 2016 to June 2018. Cervical elastography, cervical length, and endocervical canal width were measured during the second trimester in the cervical insufficiency group and control group without cervical insufficiency. Strain elastography under transvaginal ultrasound was used to assess cervical stiffness and presented as percentage (strain rate).

**Results:**

Among the 339 pregnant women enrolled, 24 had a history of cervical insufficiency and underwent cerclage. Both anterior and posterior cervical lips were significantly softer in the cervical insufficiency group even though they received cerclage (anterior strain rate: 0.18 ± 0.06% vs. 0.13 ± 0.04%; *P* = 0.001; posterior strain rate: 0.11 ± 0.03% vs. 0.09 ± 0.04%; *P* = 0.017). Cervical length was also shorter in the cervical insufficiency group (36.3 ± 3.6 mm vs. 38.3 ± 4.6 mm; *P* = 0.047). However, there was no significant difference in endocervical canal width between the two groups (5.4 ± 0.7 mm vs. 5.6 ± 0.7 mm; *P* = 0.159). Multivariate logistic regression analysis also revealed significant differences in anterior cervical lip strain rate (adjusted odds ratio [OR], 7.32, 95% confidence interval [CI], 1.70-31.41; *P* = 0.007), posterior cervical lip strain rate (adjusted OR, 5.22, 95% CI, 1.42–19.18; *P* = 0.013), and cervical length (adjusted OR, 3.17, 95% CI,1.08–9.29; *P* = 0.035). Among the four ultrasound parameters, softer anterior cervical lip (*P* = 0.024) and shorter cervical length (*P* < 0.001) were significantly related to preterm delivery.

**Conclusions:**

Cervical cerclage can prevent widening of the endocervical canal, but not improve cervical elasticity or cervical length. Measuring anterior cervical elastography and cervical length may be valuable to predict preterm delivery.

## Background

Cervical insufficiency is defined as the inability of the cervix to retain a pregnancy in the absence of uterine contractions [1]. The diagnosis is based on a history of fetal loss in the second trimester without signs of labor or uterine contractions, shortened cervical length on sonography, or painless cervical dilatation on speculum examination. Cervical insufficiency is associated with preterm birth, neonatal morbidity and mortality, and accounts for 1:100 to 1:2000 of all pregnancies [2].

Cervical insufficiency is considered to be a structural deficiency resulting in the cervix being unable to maintain its integrity, and cervical cerclage is a surgical intervention for the mechanical prevention of preterm birth which has been shown to improve neonatal morbidity and mortality [1, 3]. The two most commonly performed cerclage procedures are modifications of techniques by Shirodkar and McDonald [4, 5]. The Shirodkar technique involves dissecting the vaginal mucosa, retracting the bladder and rectum to expose the internal cervical os, and using a tape passed through the submucosal tunnel on both sides of the cervix; the McDonald technique uses a suture placed around the cervicovaginal junction in a purse-string method without dissection of the bladder or rectum. Despite the use of these techniques, these women remain at a higher risk of preterm birth [1, 6].

Previous studies have demonstrated associations between various cervical parameters measured by ultrasound with cervical insufficiency, including cervical length [1, 7], endocervical canal width [8, 9], and cervical elastography [10–12]. Elastography is a sonographic imaging technique used to assess the stiffness of tissue with strain or shear-wave methods [10]. The effects of cerclage on cervical anatomy are unclear. Previous studies have compared cervical length before and after cerclage, and some have reported an increase in cervical length after cerclage [13–16]; however, changes in cervical elastography and endocervical canal width after cerclage have not been well explored.

This study was a secondary analysis of our previous research, in which we demonstrated that pregnant women with a history of cervical insufficiency had a softer anterior cervical lip, shorter cervical length and wider endocervical canal in the first trimester [10]. In this study, we compared second-trimester cervical length, endocervical canal width and cervical elastography between pregnant women with a history of cervical insufficiency after cervical cerclage and those without cervical insufficiency. We further explored the relationship between these cervical parameters and preterm delivery.

## Methods

### Study population

This was an observational ultrasound study of singleton pregnancies at MacKay Memorial Hospital, a tertiary referral medical center in Taiwan from January 2016 to June 2018. All of the enrolled women received transvaginal sonographic examinations during the first trimester (11^+ 0/7^ to 13^+ 6/7^ gestational weeks) and second trimester (21^+ 0/7^ to 25^+ 6/7^ gestational weeks). Cervical insufficiency was confirmed based on a history of fetal loss during the second trimester without signs of labor, and our study did not include asymptomatic women who did not have a history of such second-trimester fetal loss. Gestational age was determined in the first trimester by measuring the fetal crown-rump length under sonography. The exclusion criteria were: (1) multifetal gestation, (2) first trimester vaginal bleeding, (3) previous cerclage in a prior pregnancy, (4) previous spontaneous preterm birth not because of cervical insufficiency, and (5) previous loop electrosurgical excision or cervical conization. This study was conducted retrospectively as an observational ultrasound study after the women had given birth, ensuring that their medical treatments remained unaffected by our research. To ensure a representative sample, we employed a stratified random sampling approach, stratifying participants based on age groups. This study was approved by the Institutional Review Board of MacKay Memorial Hospital (IRB no. 22MMHIS258e). All personal identifiers were anonymized prior to data collection.

### Ultrasound examinations

**Fig. 1:**
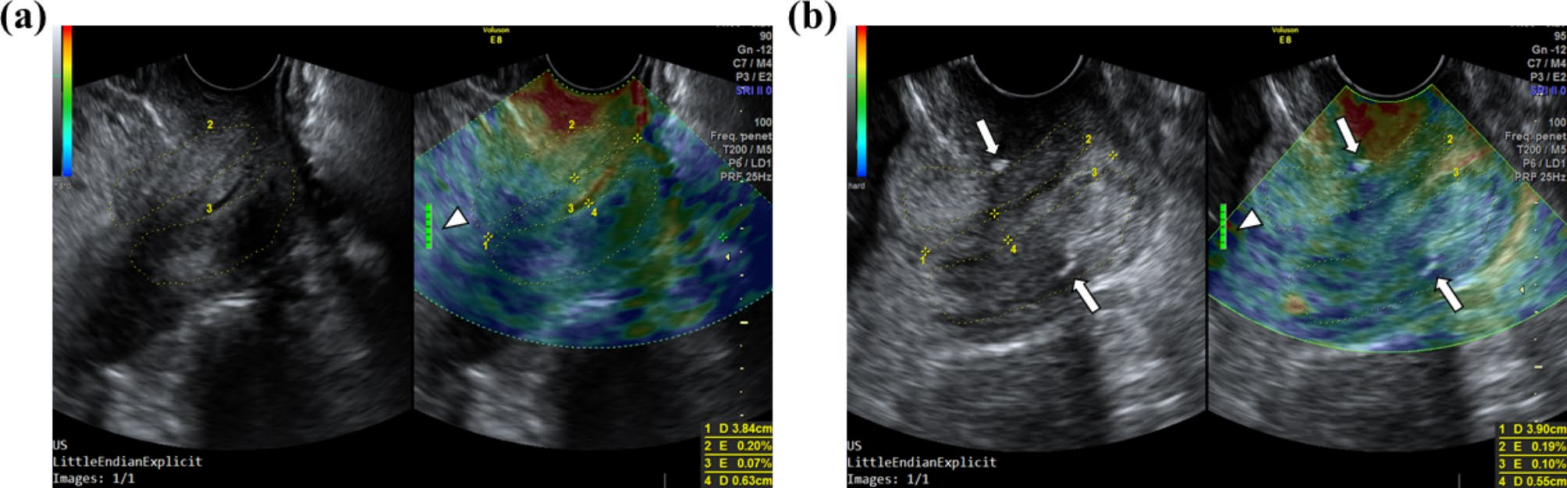
Dual-mode grayscale sonographic images (left) and real-time elastograms (right) of an insufficient cervix in the first trimester (a) and the second trimester (b). Elastograms showed differences in the stiffness of anterior and posterior cervical lips visualized as different colors, with red, blue, and green indicating soft, hard, and intermediate tissues, respectively. The cervical length and endocervical canal width were also measured. Full green quality bar was achieved after appropriate manual compression (arrowhead). Cerclage stiches are shown in the second trimester (arrow)

All ultrasound examinations were conducted using a Voluson E8 ultrasound machine (GE Medical Systems) with a 4–9 MHz transvaginal probe. During each examination, the pregnant woman was placed in the dorsal lithotomy position after bladder evacuation, and the transvaginal probe was placed in the anterior vaginal fornix. The optimal view of the cervix was mid-sagittal, permitting evaluation of both anterior and posterior cervical lips and entire endocervical canal. The interrogation box for color elastography included the entire cervix, and we shifted to dual mode (with a grayscale image and an elastogram), allowing the operator to trace the contour of the cervix under B-Mode and the region of cervical elastography at the same time (Fig. 1). Cervical length was measured first. Defined as the length of the endocervical canal, it was measured as the linear distance between two ends of the glandular area of the endocervix, which was identified as endocervical mucosa. We then measured cervical elastography. Upon applying external pressure to the anterior cervical lip, a real-time elastogram of the anterior and posterior cervical lips could be obtained. With appropriate manual pressure, an optimal measurement could be achieved as indicated by a green quality bar (Fig. 1). On color elastography, high strain was shown in red, indicating softer tissue, low strain in blue, indicating stiffer tissue, and moderate strain in green. Strain rates of the cervical lips were measured and calculated by the ultrasound machine and presented as percentages, with a higher strain rate indicating softer tissue. Finally, the endocervical canal width was measured at its widest part, which was in most cases hyperechogenic.

All ultrasound examinations in this study were performed by the same obstetrician (CY Chen), with over 25 years of ultrasound examination experience. In addition, all sonographic imaging settings were identical: Elasto map: 5; Persistence filter: 6; Line density: 1; Window length: 22; Window step: 4; Filter axial: 2; Filter lateral: 7; Frame reject: 0; Pixel reject: 0; Transparency: 200; Frequency: penetration; Pulse repetition frequency: 25 Hz. Elastograms of the cervical lips were measured twice in the first 20 cases to evaluate intra-rater reliability, which, as reported in our previous study, showed excellent agreement with intraclass correlation coefficients of the anterior and posterior cervical lips of 0.95 and 0.93, respectively [10].

### Cervical cerclage

Women with a history of cervical insufficiency underwent early second-trimester cervical cerclage. The choice between the Shirodkar and McDonald methods for cerclage was based on the operator’s discretion [10]. The Shirodkar cerclage procedure employed a 5-mm Mersilene tape (Ethicon, UK), while the McDonald cerclage procedure utilized a number 2 Ethibond (Ethicon, UK). In our study, routine administration of progesterone was not employed for women with a history of cervical insufficiency following cerclage placement.

### Statistical analysis

SPSS version 28.0 (IBM Corporation, Armonk, NY, USA) was used for all statistical analyses. The chi-square test or Fisher’s exact test was used for categorical variables, and the Student’s *t* test was used for continuous variables. Receiver operating characteristic curves and the Youden index were used to assess the optimal cut-off values of each parameter related to cervical insufficiency, and the area under the curve was derived. Univariate and multivariate logistic regression analyses were used to investigate individual parameters related to cervical insufficiency. A *P* value < 0.05 was considered statistically significant.

## Results

A total of 339 pregnant women were enrolled, of whom 24 had a history of cervical insufficiency and received cervical cerclage (21 received Shirodkar cerclage and 3 received McDonald cerclage). The mean gestational age at cerclage was 13.3 ± 0.5 weeks. Table 1 shows the maternal characteristics and neonatal outcomes. The group with cervical insufficiency had an earlier gestational age of delivery (35.8 ± 4.7 weeks vs. 38.1 ± 3.4 weeks; *P* = 0.030) and a higher rate of preterm delivery (29.2% vs. 7.9%; *P* = 0.004). In this study, 7 (29.2%) of the 24 women with a history of cervical insufficiency after cerclage were preterm delivery and the causes of preterm birth were: spontaneous preterm labor (n = 1), preterm prelabor rupture of membranes (n = 4), intrauterine infection (n = 1), and preeclampsia with severe features (n = 1). In addition, the neonates of the women with cervical insufficiency had a lower birthweight (2561.9 ± 838.5 g vs. 3003.3 ± 559.7 g; *P* = 0.018).

**Table 1:** Maternal characteristics and neonatal outcomes

	Cervical insufficiency (n = 24)	No cervical insufficiency (n = 315)	*P* value
Mother			
Age (year)	32.6 ± 4.1	32.0 ± 4.0	0.437
BMI (kg/m^2^)	27.6 ± 4.9	25.5 ± 3.6	0.006*
Gravida	3.1 ± 1.1	1.90 ± 1.0	< 0.001*
Para	1.4 ± 1.0	0.8 ± 0.	< 0.001*
Cesarean section	8 (33.3)	73 (28.0)	0.577
Delivery age (week)	35.8 ± 4.7	38.1 ± 3.4	0.030*
Preterm delivery	7 (29.2)	25 (7.9)	0.004*
Neonate			
Birth age (week)	35.8 ± 4.7	38.1 ± 3.4	0.030*
Birth weight (g)	2561.9 ± 838.5	3003.3 ± 559.7	0.018*
Apgar score			
1 min	8.2 ± 2.2	8.9 ± 1.6	0.058
5 min	9.0 ± 2.0	9.5 ± 1.4	0.120

Continuous variables are presented as mean ± standard deviation and categorical variables as n (%)

BMI: body mass index

*P < 0.05 was considered statistically significant

**Fig. 2:**
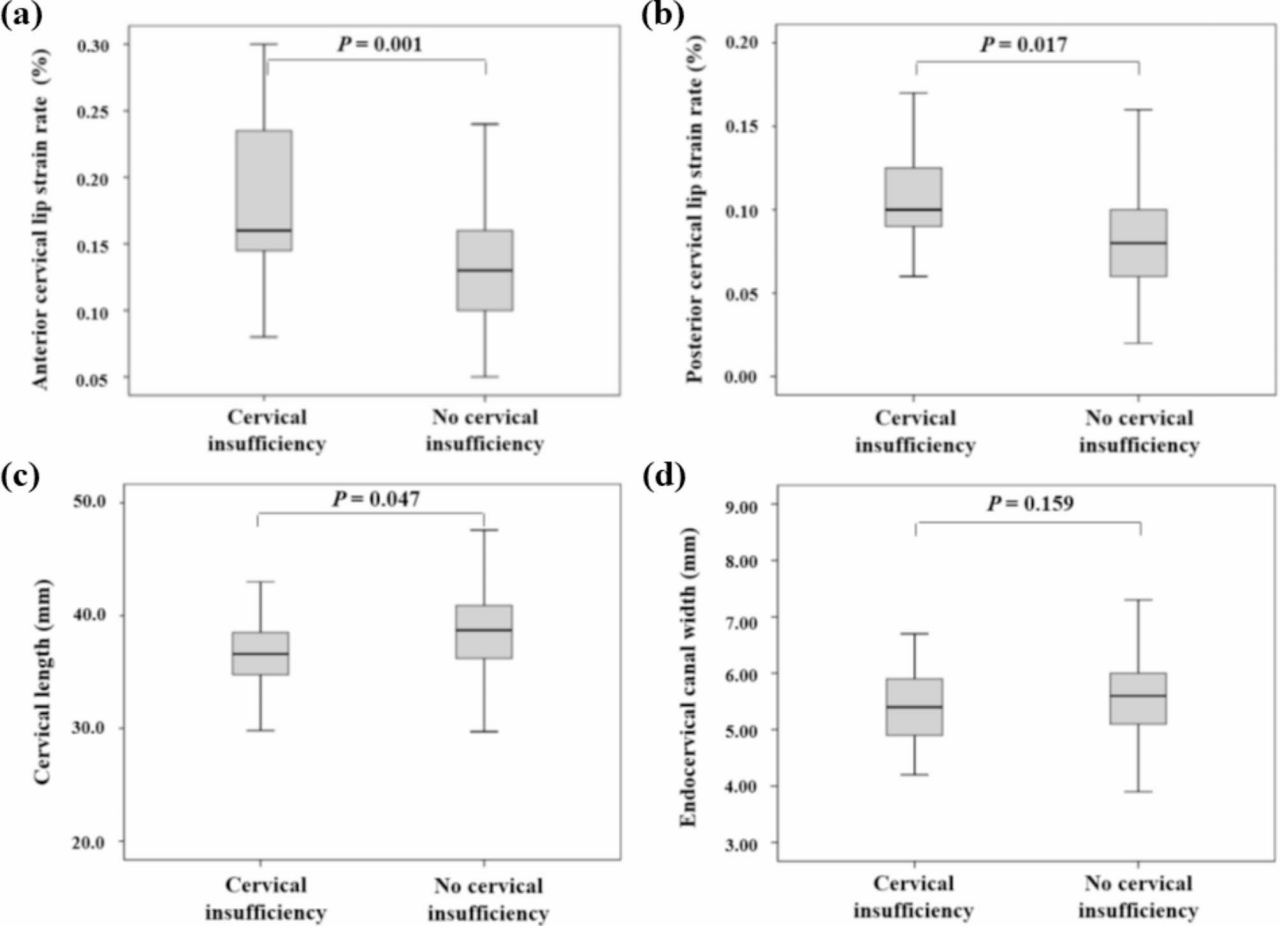
Box-and-whisker plots of (**a**) anterior cervical lip strain rate, (**b**) posterior cervical lip strain rate, (**c**) cervical length, and (**d**) endocervical canal width between the cervical insufficiency and no cervical insufficiency groups. Boxes show median and interquartile range, and whiskers represent the 5th and 95th percentiles

Table 2 shows comparisons of cervical elastography, cervical length and endocervical canal width between the cervical insufficiency group and control group during the first and second trimesters. In the first trimester scan, the cervical insufficiency group had a softer anterior cervical lip (strain rate: 0.19 ± 0.05% vs. 0.11 ± 0.04%; *P* < 0.001), shorter cervical length (36.3 ± 4.8 mm vs. 38.3 ± 3.8 mm; *P* = 0.014), and wider endocervical canal width (5.7 ± 1.1 mm vs. 5.2 ± 0.7 mm; *P* = 0.001). However, posterior cervical lip elasticity was not significantly different between the two groups in the first trimester. In the second trimester scan, the cervical insufficiency group after cerclage had a softer anterior cervical lip (strain rate: 0.18 ± 0.06% vs. 0.13 ± 0.04%; *P* = 0.001), softer posterior cervical lip (strain rate: 0.11 ± 0.03% vs. 0.09 ± 0.04%; *P* = 0.017), and shorter cervical length (36.3 ± 3.6 mm vs. 38.3 ± 4.6 mm; *P* = 0.047). Nevertheless, the endocervical canal width was not significantly different between the cervical insufficiency group after cerclage and the control group in the second trimester. Box-and-whisker plots revealed that medians of second-trimester anterior, posterior cervical strain rates, and cervical length were significantly different between the two groups, but that there was no significant difference in endocervical canal width (Fig. 2).

**Table 2:** Cervical elastography, cervical length, and endocervical canal width between the study and control groups

	Cervical insufficiency (n = 24)	No cervical insufficiency (n = 315)	*P* value
First trimester			
GA at scan (week)	12.6 ± 0.6	12.5 ± 0.5	0.445
CRL (mm)	69.3 ± 7.4	67.9 ± 6.1	0.259
Strain rate (%)			
Anterior cervical lip	0.19 ± 0.05	0.11 ± 0.04	< 0.001*
Posterior cervical lip	0.09 ± 0.05	0.08 ± 0.07	0.690
Cervical length (mm)	36.3 ± 4.8	38.3 ± 3.8	0.014*
Endocervical canal width (mm)	5.7 ± 1.1	5.2 ± 0.7	0.001*
Second trimester			
GA at scan (week)	21.9 ± 0.5	22.0 ± 0.5	0.507
BPD (mm)	54.7 ± 2.1	54.6 ± 2.2	0.759
AC (mm)	175.8 ± 6.3	176.4 ± 7.7	0.728
FL (mm)	37.4 ± 2.2	37.9 ± 1.6	0.166
Strain rate (%)			
Anterior cervical lip	0.18 ± 0.06	0.13 ± 0.04	0.001*
Posterior cervical lip	0.11 ± 0.03	0.09 ± 0.04	0.017*
Cervical length (mm)	36.3 ± 3.6	38.3 ± 4.6	0.047*
Endocervical canal width (mm)	5.4 ± 0.7	5.6 ± 0.7	0.159

Continuous variables are presented as mean ± standard deviation

GA: gestational age; CRL: crown-lump length; BPD, biparietal diameter; AC, abdominal circumference; FL, femoral length

*P < 0.05 was considered statistically significant

**Fig. 3:**
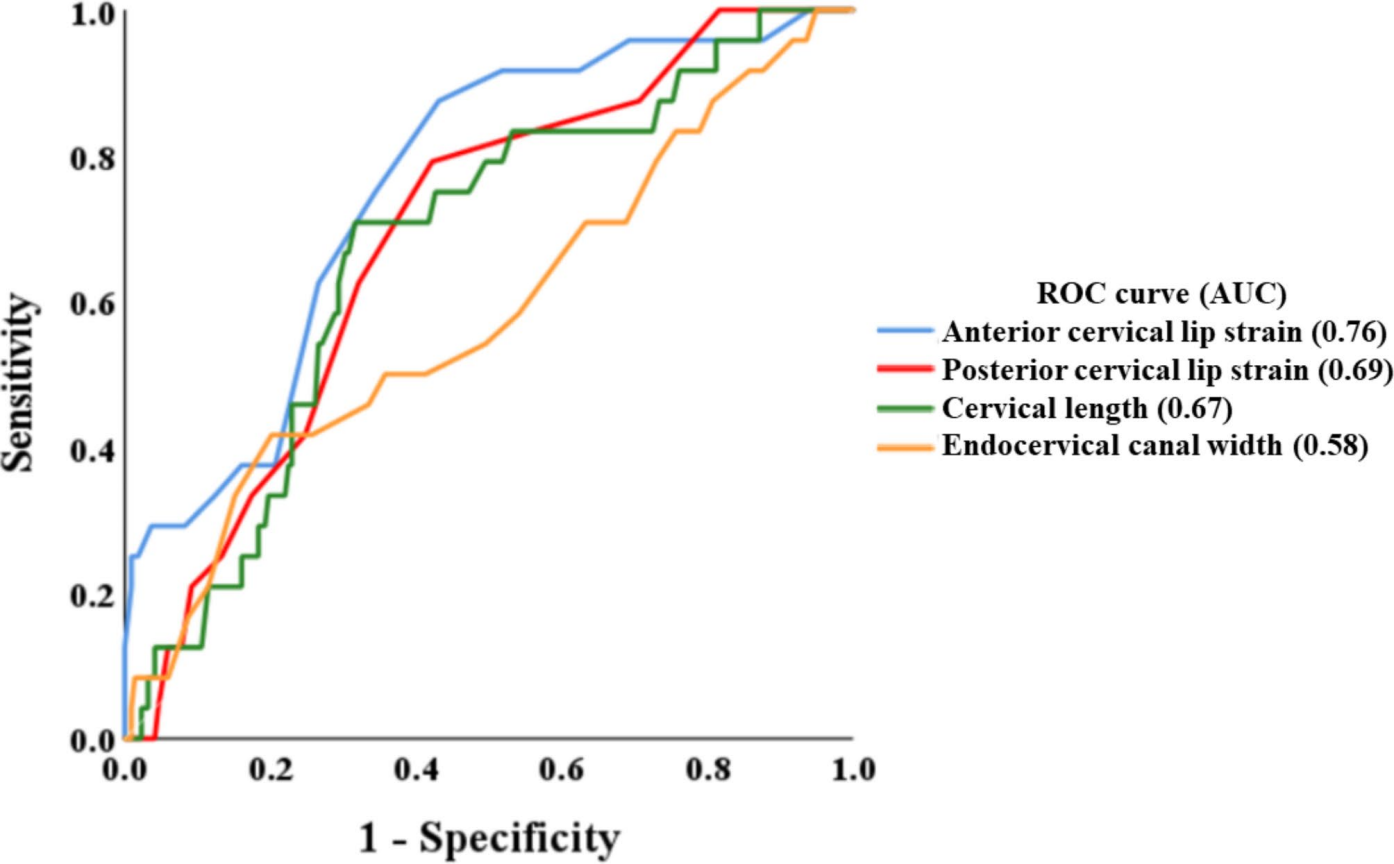
Receiver operating characteristic (ROC) curves and the areas under the curves (AUCs) of anterior cervical lip strain, posterior cervical lip strain, cervical length, and endocervical canal width to predict cervical insufficiency. The AUCs for anterior cervical lip strain, posterior cervical lip strain, cervical length, and endocervical canal were 0.76, 0.69, 0.67, and 0.58, respectively

Table 3; Fig. 3 show the results of receiver operating characteristic curve analyses of second-trimester parameters related to cervical insufficiency. The cut-off values of second-trimester anterior, posterior cervical strain rates, and cervical length to confirm women with a history of cervical insufficiency were 0.14%, 0.09%, and 37.4 mm, respectively. The areas under the curves of second-trimester anterior, posterior cervical strain rates, and cervical length were 0.76, 0.69, and 0.67, respectively. Multivariate logistic regression analyses also revealed significant differences in anterior cervical lip strain rate (adjusted odds ratio [OR], 7.32, 95% confidence interval [CI], 1.70-31.41; *P* = 0.007), posterior cervical lip strain rate (adjusted OR, 5.22, 95% CI, 1.42–19.18; *P* = 0.013), and cervical length (adjusted OR, 3.17, 95% CI, 1.08–9.29; *P* = 0.035) (Table 4).

**Table 3:** ROC curve analyses of second-trimester cervical elastography, cervical length and endocervical canal width for the diagnosis of cervical insufficiency

	Cut-off value	Sensitivity	Specificity	AUC	95% CI	*P* value
Anterior cervical lip strain rate	0.14%	0.88	0.55	0.76	0.66–0.85	< 0.001*
Posterior cervical lip strain rate	0.09%	0.79	0.58	0.69	0.59–0.79	0.002*
Cervical length	37.4 mm	0.66	0.71	0.67	0.57–0.78	0.005*
Endocervical canal width				0.58	0.46–0.71	0.186

ROC, receiver operating characteristic; AUC, area under the curve; CI, confidence interval

*P < 0.05 was considered statistically significant

**Table 4:** Univariate and multivariate logistic regression analyses of individual parameters related to cervical insufficiency

Parameter	Univariate logistic regression			Multivariate logistic regression			
	Unadjusted OR	95% CI	*P* value	Adjusted OR	95% CI		*P* value
Age	1.04	0.94–1.16	0.436				
BMI	1.14	1.03–1.25	0.008*	1.14	0.99–1.31	0.077	
Gravida	2.27	1.61–3.21	< 0.001*	2.62	1.57–4.39	< 0.001*	
Para	2.22	1.40–3.51	0.001*	1.30	0.62–2.72	0.491	
BPD	1.35	0.20–9.27	0.758				
AC	0.90	0.51–1.60	0.727				
FL	0.15	0.01–2.16	0.162				
s-trimester strain rate							
Anterior cervical lip							
≥ 0.14%	8.66	2.51–29.87	0.001*	7.32	1.70–31.41	0.007*	
< 0.14%	1			1			
Posterior cervical lip							
≥ 0.09%	5.15	1.86–14.27	0.002*	5.22	1.42–19.18	0.013*	
< 0.09%	1			1			
s-trimester cervical length							
≤ 37.4 mm	4.79	1.91–12.06	0.001*	3.17	1.08–9.29	0.035*	
> 37.4 mm	1			1			
Second-trimester endocervical canal width	0.01	0.00–6.20	0.157				

BMI: body mass index; BPD, biparietal diameter; AC, abdominal circumference; FL, femoral length; OR: odds ratio; CI: confidence interval

*P < 0.05 was considered statistically significant

**Fig. 4:**
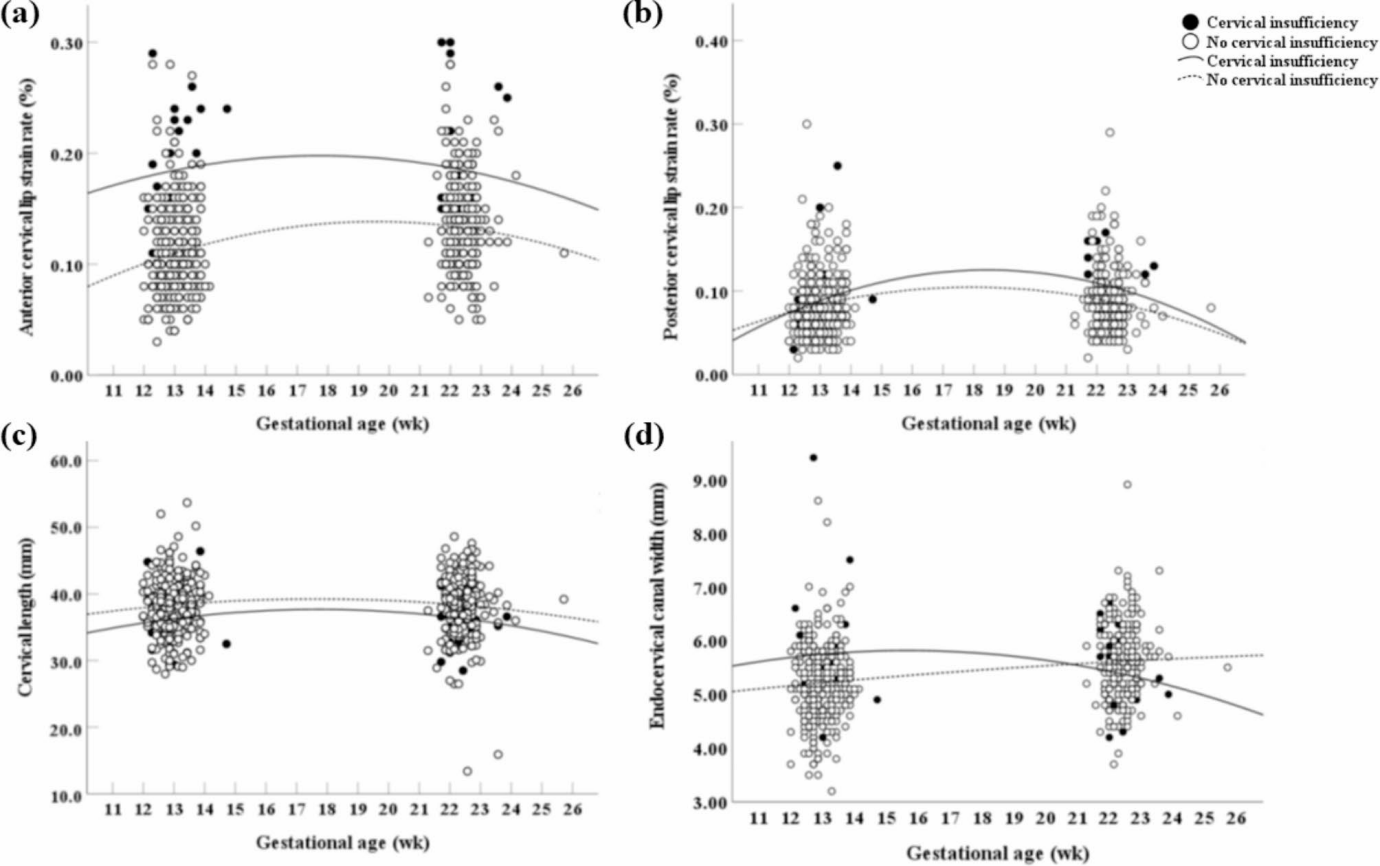
Comparisons of (**a**) anterior cervical lip strain rate, (**b**) posterior cervical lip strain rate, (**c**) cervical length, and (**d**) endocervical canal width between the cervical insufficiency and no cervical insufficiency groups according to gestational age

Figure 4 shows changes in the four parameters between the cases and controls according to gestational age. The patterns of changes in cervical length, anterior, and posterior cervical lip strain rates were similar in both groups throughout gestation, even though cervical cerclage was performed in the cervical insufficiency group. Only the pattern of change in endocervical canal width was different between the two groups, and it increased over time in the women without cervical insufficiency, while it decreased in the women with cervical insufficiency after cervical cerclage.

Table 5 shows the correlations between second-trimester parameters and preterm delivery. Among the four ultrasound parameters, softer anterior cervical lips (strain rate: 0.15 ± 0.07% vs. 0.14 ± 0.04%; *P* = 0.024) and shorter cervical length (37.1 ± 6.9 mm vs. 38.2 ± 4.2 mm; *P* < 0.001) were significantly related to preterm delivery.

**Table 5:** Correlations between second-trimester parameters and preterm delivery

	Preterm delivery (n = 32)	Term delivery (n = 307)	*P* value
Anterior cervical lip strain rate (%)	0.15 ± 0.07	0.14 ± 0.04	0.024*
Posterior cervical lip strain rate (%)	0.09 ± 0.04	0.09 ± 0.04	0.891
Cervical length (mm)	37.1 ± 6.9	38.2 ± 4.2	< 0.001*
Endocervical canal width (mm)	5.7 ± 0.8	5.6 ± 0.7	0.922

*P < 0.05 was considered statistically significant

## Discussion

In our previous study, we demonstrated that pregnant women with a history of cervical insufficiency had a softer anterior cervical lip, shorter cervical length and wider endocervical canal in the first trimester [10]. In this study, we further compared changes in cervical elastography, cervical length, and endocervical canal width after cerclage in pregnant women with cervical insufficiency after cerclage and in those without cervical insufficiency in the second trimester. Significant differences in cervical elastography and cervical length were found between the cases and controls, however there was no significant difference in endocervical canal width between the two groups, suggesting that cerclage could effectively decrease endocervical canal width.

Cervical elastography showed that the strain rate of anterior cervical lips was higher than that of posterior cervical lips. It is reasonable to assume that the anterior cervical lip is closer to the ultrasound transducer and therefore is subject to more compression force. In this study, both anterior and posterior cervical lips remained softer in the cervical insufficiency group during the second trimester, whereas only the anterior cervical lip was softer in the first trimester in our previous study [10]. Louwagie et al. demonstrated that the anterior uterocervical angle shifts posteriorly throughout gestation using biomechanical solid modeling [17]. Multiple factors contribute to cervical softening, including progressive changes in cervical tissue hydration, collagen structure and tissue elasticity [18]. Our results demonstrated that cerclage could not effectively improve cervical softness.

Several studies have compared cervical length before and after prophylactic or therapeutic cerclage, and some have demonstrated that cervical length may increase after cerclage [13–16]. Correlations between increased cervical length and decreased preterm birth in previous studies have been inconsistent, however progressive cervical shortening or a static cervical length after cerclage has been reported to be predictive of preterm delivery [15, 16]. In our study, the mean cervical length did not significantly change after cerclage, and the second-trimester cervical length in the cervical insufficiency group was significantly shorter and the preterm birth rate was also higher than in the control group.

Previous studies have reported correlations between a wider endocervical canal and cervical insufficiency [8, 9]. In our previous study, we also demonstrated a wider endocervical canal in women with cervical insufficiency during the first trimester [10]. After cerclage, there was no significant difference in endocervical canal width between the cases and controls in the second trimester. In the present study, we also found that the endocervical canal width increased with gestation in the control group but decreased in the cervical insufficiency group after cerclage. Research related to changes in endocervical canal width among patients after cerclage is limited. Azar et al. reported that the mean cervical width significantly decreased after elective or urgent cerclage, which is consistent with our results [19]. Cerclage placement is used to reinforce the cervix and provide it with a physical support to prevent widening of the endocervical canal. This could be the main mechanism by which cerclage prolongs gestation and improves pregnancy outcome.

Cervical softening in women with cervical insufficiency is a complex process involving histological, mechanical, and biochemical changes of the extracellular matrix, mainly of collagen, and multiple pathophysiologic pathways are associated with cervical shortening and dilatation resulting in preterm birth [18, 20]. Iams et al. reported that cervical insufficiency was not a categorical variable but rather a continuum [7]. Therefore, the procedure of cerclage alone cannot improve all causes of an incompetent cervix. These mechanisms may also explain the reported benefits of adjuvant progesterone treatment after cerclage to prevent preterm delivery in previous research [21–23]. Progesterone can decrease prostaglandin synthesis, inhibit cervical stromal degradation, and decrease myometrial activation, which may enhance the effect of cerclage [24].

Despite the use of cerclage placement for women with cervical insufficiency, they are still at a higher risk of preterm birth [1, 6]. Measuring the cervical length in the second trimester has been demonstrated to be able to identify women at an increased risk of preterm delivery [25, 26]. In addition, cervical elastography in the second trimester has recently been used to predict preterm delivery with promising results [27–29]. Our results showed that shorter cervical length and softer anterior cervical lip were associated with preterm delivery, which is consistent with previous studies. As mentioned earlier, cervical softening entails intricate alterations in the extracellular matrix, encompassing histological, mechanical, and biochemical changes [18, 20]. Elastography offers a means to assess cervical tissue quality by measuring its stiffness. In contrast, cervical length measurements predominantly concentrate on the cervix’s physical dimensions. Given that preterm birth is frequently linked to shifts in cervical tissue composition, elastography provides a more direct evaluation of cervical tissue health. Consequently, the assessment of cervical elasticity has the potential to improve the precision of preterm birth risk prediction, surpassing the reliance solely on cervical length measurements.

The strengths of this study include that we investigated three different characteristics of the cervix after cerclage in the second trimester at a single institution, thereby reducing procedural differences. In addition, we performed elastography of the entire cervix, including anterior and posterior cervical lips, which could reduce possible measurement bias if investigating only part of the cervix. However, there are still several limitations. First, cervical strain elastography depended on the pressure of the transvaginal probe that the examiner applied to the cervix, which is difficult to standardize, although all elastograms were measured by the same examiner. Second, the examiner was not blinded to the obstetric history and cerclage placement of the study group, which may have influenced the results of ultrasound measurements. Third, our study was unable to establish the optimal timeframe for predicting the efficacy of cerclage placement since we did not conduct consecutive cervical examinations. Further research with consecutive post-cerclage cervical examinations is required to clarify this timeframe. Finally, our study was unable to determine whether changes in elastography among women with a history of cervical insufficiency are linked to a predisposition to preterm birth or are a result of preterm birth, for which a further prospective study is indicated.

## Conclusion

Our results showed that cervical cerclage could effectively prevent widening of the endocervical canal, but not improve cervical elasticity or cervical length. These findings may offer insights into the reported benefits of adjuvant progesterone treatment following cerclage in preventing preterm delivery. They also explain that women with cerclage placement still experience an increased risk of preterm birth. Knowing these changes of the cervix after cerclage may help to develop new strategies to prevent preterm delivery in women with cervical insufficiency. We also found a negative correlation between cervical length and preterm delivery, and a positive correlation between anterior cervical lip strain and preterm delivery. Beyond cervical length, cervical elastography could be an alternative ultrasound cervical assessment tool to predict preterm delivery.

## Data Availability

Data have been collected at MacKay Memorial Hospital, a tertiary referral medical center in Taiwan. The datasets used and/or analyzed during the current study are available from the corresponding author on reasonable request.

## Abbreviations

OR: Odds ratio
CI: Confidence interval

## References

[1] American College of Obstetricians and Gynecologists (2014). ACOG Practice Bulletin No.142: Cerclage for the management of cervical insufficiency. Obstet Gynecol.

[2] Lidegaard O (1994). Cervical incompetence and cerclage in Denmark 1980–1990. A register based epidemiological survey. Acta Obstet Gynecol Scand.

[3] Boelig RC, Berghella V (2017). Current options for mechanical prevention of preterm birth. Semin Perinatol.

[4] Shirodkar VN (1955). A new method of operative treatment for habitual abortion in the second trimester of pregnancy. Antiseptic.

[5] McDonald IA (1957). Suture of the cervix for inevitable miscarriage. J Obstet Gynaecol Br Emp.

[6] Suhag A, Berghella V (2014). Cervical cerclage. Clin Obstet Gynecol.

[7] Iams JD, Johnson FF, Sonek J, Sachs L, Gebauer C, Samuels P (1995). Cervical competence as a continuum: a study of ultrasonographic cervical length and obstetric performance. Am J Obstet Gynecol.

[8] Varma TR, Patel RH, Pillai U (1986). Ultrasonic assessment of cervix in ‘at risk’ patients. Acta Obstet Gynecol Scand.

[9] Podobnik M, Bulić M, Smiljanić N, Bistricki J (1988). Ultrasonography in the detection of cervical incompetency. J Clin Ultrasound.

[10] Chen CY, Chen CP, Sun FJ (2020). Assessment of the cervix in pregnant women with a history of cervical insufficiency during the first trimester using elastography. Acta Obstet Gynecol Scand.

[11] Öcal FD, Çekmez Y, Erdoğdu E (2015). The utility of cervical elastosonography in prediction of cervical insufficiency: cervical elastosonography and cervical insufficiency. J Matern Fetal Neonatal Med.

[12] Zhang L, Zheng Q, Xie H, Du L, Wu L, Lin M (2020). Quantitative cervical elastography: a new approach of cervical insufficiency prediction. Arch Gynecol Obstet.

[13] Funai EF, Paidas MJ, Rebarber A, O’Neill L, Rosen T, Young BK (1999). Change in cervical length after prophylactic cerclage. Obstet Gynecol.

[14] Althuisius SM, Dekker GA, van Geijn HP, Hummel P (1999). The effect of therapeutic McDonald cerclage on cervical length as assessed by transvaginal ultrasonography. Am J Obstet Gynecol.

[15] Dijkstra K, Funai EF, O’Neill L, Rebarber A, Paidas MJ, Young BK (2000). Change in cervical length after cerclage as a predictor of preterm delivery. Obstet Gynecol.

[16] Cook JR, Chatfield S, Chandiramani M (2017). Cerclage position, cervical length and preterm delivery in women undergoing ultrasound indicated cervical cerclage: a retrospective cohort study. PLoS ONE.

[17] Louwagie EM, Carlson L, Over V (2021). Longitudinal ultrasonic dimensions and parametric solid models of the gravid uterus and cervix. PLoS ONE.

[18] Feltovich H, Hall TJ, Berghella V (2012). Beyond cervical length: emerging technologies for assessing the pregnant cervix. Am J Obstet Gynecol.

[19] Azar ZF, Hakimi P, Ghojazadeh M, Ghatresamani F (2011). Pre- and post-McDonald cerclage cervical length, width and funneling rate and their association with duration of pregnancy. Pak J Biol Sci.

[20] Schlembach D, Mackay L, Shi L, Maner WL, Garfield RE, Maul H (2009). Cervical ripening and insufficiency: from biochemical and molecular studies to in vivo clinical examination. Eur J Obstet Gynecol Reprod Biol.

[21] Jung EY, Oh KJ, Hong JS, Han BR, Joo JK (2016). Addition of adjuvant progesterone to physical-exam-indicated cervical cerclage to prevent preterm birth. J Obstet Gynaecol Res.

[22] Roman AR, Da Silva Costa F, Araujo Júnior E, Sheehan PM (2018). Rescue adjuvant vaginal progesterone may improve outcomes in cervical cerclage failure. Geburtshilfe Frauenheilkd.

[23] Shor S, Zimerman A, Maymon R (2021). Combined therapy with vaginal progesterone, Arabin cervical pessary and cervical cerclage to prevent preterm delivery in high-risk women. J Matern Fetal Neonatal Med.

[24] Di Renzo GC, Tosto V, Tsibizova V, Fonseca E (2021). Prevention of Preterm Birth with Progesterone. J Clin Med.

[25] American College of Obstetricians and Gynecologists (2021). ACOG Practice Bulletin No.234: prediction and prevention of spontaneous preterm birth. Obstet Gynecol.

[26] Coutinho CM, Sotiriadis A, Odibo A (2022). ISUOG Practice guidelines: role of ultrasound in the prediction of spontaneous preterm birth. Ultrasound Obstet Gynecol.

[27] Patberg ET, Wells M, Vahanian SA et al. Use of cervical elastography at 18 to 22 weeks’ gestation in the prediction of spontaneous preterm birth. Am J Obstet Gynecol. 2021;225:525.e1-525.e9. 10.1016/j.ajog.2021.05.017 34051170

[28] Yang X, Ding Y, Mei J (2022). Second-trimester cervical shear wave elastography combined with cervical length for the prediction of spontaneous preterm birth. Ultrasound Med Biol.

[29] Debring B, Möllers M, Köster HA, et al. Cervical strain elastography: pattern analysis and cervical sliding sign in preterm and control pregnancies. J Perinat Med. 2022 Aug;16. 10.1515/jpm-2022-0166. Epub ahead of print. 10.1515/jpm-2022-0166 35969418

